# Enhancing maize phosphorus uptake with optimal blends of high and low-concentration phosphorus fertilizers

**DOI:** 10.3389/fpls.2024.1451073

**Published:** 2024-08-14

**Authors:** Chen Chen, Yue Xiang, Xiaoqiang Jiao, Haiqing Gong

**Affiliations:** ^1^ Ministry of Education Key Laboratory for Biodiversity Science and Ecological Engineering, National Observations and Research Station for Wetland Ecosystems of the Yangtze Estuary, College of Life Sciences, Fudan University, Shanghai, China; ^2^ College of Resources and Environmental Sciences, National Academy of Agriculture Green Development, Key Laboratory of Plant-Soil Interactions, Ministry of Education, Key Laboratory of Low-carbon Green Agriculture, Ministry of Agriculture and Rural Affairs, China Agricultural University, Beijing, China

**Keywords:** phosphorus, maize, inorganic phosphorus pool, alkaline soil, acidic soil

## Abstract

High-concentration phosphorus (P) fertilizers are crucial for crop growth. However, fertilizers with lower P concentrations, such as calcium magnesium phosphate (CMP) and single super phosphate (SSP), can also serve as efficient P sources, especially when blended with high-concentration P fertilizers like diammonium phosphate (DAP) or monoammonium phosphate (MAP). In this study, we conducted a 48-day pot experiment to explore how blending low-P fertilizers could optimize maize P utilization, using CMP to replace DAP in acidic soil, and SSP to replace MAP in alkaline soil, with five SSP+MAP and CMP+DAP mixtures tested. Key metrics such as shoot and root biomass, shoot P uptake, root length, and soil P bioavailability were measured. We found that maize biomass and P uptake with 100% DAP were comparable to those with 50% CMP and 50% DAP in acidic soil. Similar results were observed for 100% MAP compared to 50% SSP and 50% DAP in alkaline soil. Root biomass and length were largest with 100% MAP in acidic soil and at 100% DAP in alkaline soil, with no significant differences at 50% SSP or CMP substitutions for MAP and DAP, respectively. Furthermore, 50% SSP or CMP substitutions for MAP and DAP increased the content and proportion of the labile inorganic P (Pi) pool (H_2_O-Pi and NaHCO_3_-Pi), had a direct and positive effect on Olsen-P. Our findings reveal that 1:1 blends of SSP and MAP in acidic soil, and CMP and DAP in alkaline soil, effectively meet maize’s P requirements without relying solely on high-concentration P fertilizers. This indicates that strategic blending of fertilizers can optimize P use, which is crucial for sustainable agriculture.

## 1 Introduction

Phosphorus (P) is an essential limiting nutrient for crop yields in cropping systems (Raymond et al., 2021; Janes-Bassett et al., 2022). P fertilizers have been used to increase plant-available soil P concentrations, enhance crop yields, and develop P fertilizer management strategies to achieve optimal P fertilizer inputs (Gong et al., 2022a; Yan et al., 2022). High-concentration P fertilizers [i.e., monoammonium phosphate (MAP) and diammonium phosphate (DAP)] can readily provide P to soil solutions for plant uptake, owing to their high-water solubilities (Chien et al., 2011). However, previous studies have shown that high P solubility is not always necessary for crop production (Chien et al., 2009). Our previous findings also suggested that fertilizers with low P concentrations can be incorporated into fertilizer blends to improve P use efficiency (Gong et al., 2022a). Researchers have attempted to determine a suitable ratio of low- to high-P fertilizers for crop production. For instance, significant enhancement in P uptake was observed when a mixture of DAP and phosphate rock was applied at a 1:4 P ratio (Bindraban et al., 2020). Wheat P uptake was the highest under 20:80 and 10:90 struvite–DAP blend applications compared with those under 100% struvite and 100% DAP applications, indicating that the optimal fertilizer blend contained less struvite than high water-soluble DAP or MAP (Talboys et al., 2016). Despite these efforts, the suitable ratios of low- to high-P fertilizers to optimize P use and promote plant growth remain unclear. Our research aims to fill this gap by investigating the optimal blend ratios of low- and high-P fertilizers to maximize P use efficiency.

The high concentration P in DAP and MAP has high bioavailability, which can stimulate early root development and seedling growth (Nziguheba et al., 2016; Gong et al., 2022b), and subsequently enhancing enhance the uptake of the acidulated low-concentration P fertilizer (Geissler et al., 2019; Weeks et al., 2023). This P supply over time ensures a continuous supply throughout the plant growth cycle. The transformation of soil P involves significant physicochemical changes, including dissolution, precipitation, adsorption, and desorption (Ghodszad et al., 2022; Zheng et al., 2023), contributing to the complex and dynamic equilibrium of different forms of soil P. Fractionated soil P analysis is typically utilized to understand this equilibrium, helping to evaluate the interconversion among various soil P fractions that reflect the different P pools present in the soil (Kim et al., 2023; Zhang et al., 2020). Soil P availability is expected to increase due to the transformation of stable P into labile P forms during early root development and the subsequent application of acidulated low-concentration P fertilizers (Chen et al., 2021; Joshi et al., 2021). Further research is needed to explore how low- and high-concentration P fertilizer blends interact with different soil P fractions under optimal ratios for crop production. This may lead to more accurate predictions of soil P bioavailability and improve soil P management strategies.

Previous studies have largely depended on mineral P, particularly in high-concentration P fertilizers such as DAP and MAP, to formulate P fertilizer management strategies, however, this reliance may lead to low P use efficiency, and an increased risk of P loss and aquatic environmental pollution (Bindraban et al., 2020; Weeks and Hettiarachchi, 2019). Therefore, it is crucial to develop strategies for managing high-concentration P fertilizers. One effective approach involves blending a high-concentration P fertilizer (e.g., MAP or DAP) with a lower P-concentration fertilizer. This strategy aims to provide sufficient early-season P while reducing pollution risks associated with the rapid solubilization of high-concentration P fertilizers (Everaert et al., 2018), thereby improving P use efficiency. By quantifying crop-specific responses to the proportion of low-P fertilizer in the blend could identify the ratios of low- and high-P fertilizers that maximize crop growth potential. Therefore, the objectives of this pot experiment were to: (i) quantify the effects of different ratios of low- and high-P fertilizer blends on maize biomass and P uptake in soils with different pH levels to determine the optimal ratios for the fertilizer blends; (ii) determine the effects of the optimal fertilizer blends on soil P pool transformation; and (iii) explore potential approaches in which low-P fertilizer is used effectively in fertilizer blends to improve maize P uptake. Our findings can serve as a reference for developing efficient P fertilizer management strategies that ensure optimal use of P resources.

## 2 Materials and methods

### 2.1 Experimental setup and design

A pot experiment using maize was conducted from May to July 2022 in a greenhouse at the Quzhou Experimental Station of China Agricultural University in Quzhou County, Hebei Province, China. Alkaline soil was collected from Quzhou (Hebei Province), the basic physical and chemical properties of the soil were as follows: pH, 8.13 (in water); soil organic carbon content, 9.69 g kg^-1^; total nitrogen content, 1.10 g kg^-1^; soil Olsen-P content, 2.49 mg kg^-1^; and soil available potassium content, 183 mg kg^-1^. Acidic red soil was collected from Yuxi (Yunnan Province), the basic physical and chemical properties of the soil were as follows: pH, 5.34 (in water); soil organic carbon content, 3.85 g kg^-1^; total nitrogen content, 0.80 g kg^-1^; soil Olsen-P content, 1.42 mg kg^-1^; and soil available potassium content, 153 mg kg^-1^. Both soil types were air-dried and sieved through a 2-mm mesh.

One control group and five treatment groups were established for the acidic red soil: no P (CK), 100% calcium magnesium phosphate (100% CMP), 30% CMP and 70% DAP (30% CMP+70% DAP), 50% CMP and 50% DAP (50% CMP+50% DAP), 70% CMP and 30% DAP (70% CMP+30% DAP), and 100% DAP (100% DAP). Similarly, a control and five treatment groups were established for the alkaline soil: no P (CK), 100% single superphosphate (100% SSP), 30% SSP and 70% MAP (30% SSP+70% MAP), 50% SSP and 50% MAP (50% SSP+50% MAP), 70% SSP and 30% MAP (70% SSP+30% MAP), and 100% MAP (100% MAP). Each of the six treatments had four replicates (for a total of 48 pots), and the pots were arranged based on a completely randomized design. Each pot received 150 kg P ha^-1^ of the corresponding fertilizer blend. In the treatments that received fertilizer, urea, and muriate of potash were applied as nitrogen and potassium sources, respectively. Both nitrogen (N) and potassium (K₂O ) were applied at a rate of 150 kg ha^-1^ (**Table 1**). All fertilizers were incorporated and mixed completely with the soil during planting.

**Table 1:** Fertilization treatments applied in the present study.

Soil	Treatment	Nitrogen (kg N ha^-1^)	Phosphorus (kg P ha^-1^)	Potassium kg K₂O ha^-1^
Acidic soil	CK	150	0	150
	100% CMP	150	150	150
	70% CMP+30% DAP	150	150	150
	50% CMP+50% DAP	150	150	150
	30% CMP+70% DAP	150	150	150
	100% DAP	150	150	150
Alkaline soil	CK	150	0	150
	100% SSP	150	150	150
	70% SSP+30% MAP	150	150	150
	50% SSP+50% MAP	150	150	150
	30% SSP+70% MAP	150	150	150
	100% MAP	150	150	150

Maize seeds (*Zea mays L*. cv. Zhengdan958) were surface sterilized using 10% (v/v) H_2_O_2_ for 30 min, washed three times with deionized water, soaked in a supersaturated calcium sulfate (CaSO_4_) solution for 1 d, and germinated in a dish lined with wet filter paper and covered with an aerated cover for 3 d at 25°C. Uniformly germinated seeds were selected and sown in the pots. The pots were watered daily to 80% field capacity as measured by weight. The temperature in the glasshouse ranged from 20°C at night to 30°C during the day.

### 2.2 Plant harvesting and sample analysis

The maize plants were harvested after eight weeks. After separating the shoots and roots, the shoots were oven-dried at 105°C for 0.5 h and then dried at 75°C for 4 d to a constant weight. The dry samples were weighed, crushed, and homogenized. Each fraction was dried, and the milled plant samples were digested in a mixture of sulfuric acid and hydrogen peroxide (H_2_SO_4_-H_2_O_2_) as described by Wolf (1982). The P concentrations of the digested samples were determined using the standard vanadomolybdate method (Murphy and Riley, 1962). Shoot P uptake was calculated using the dry weights and P concentrations of the different plant parts.

The roots were carefully separated from the soil and shaken to remove any excess soil. All visible roots in each pot were collected from the soil onto a 2 mm diameter mesh and washed with running water in the lab until they were clean. The root samples were spread onto a transparent tray with water and scanned using an EPSON scanner at 400 dpi (Epson Expression 1600 pro, Model EU-35, Japan). The total root lengths were calculated from the images using the WinRHIZO software package (Pro2004b, version 5.0; Regent Instruments Inc., QC, Canada). The roots were then oven-dried and weighed to obtain the biomass.

The soil samples were dried in the open air and sifted through a 2-mm-mesh sieve. After sample digestion with H_2_SO_4_ and perchloric acid (HClO_4_), the soil total P was determined using an ultraviolet spectrometer (UV 2500, Shimadzu, Tokyo, Japan). The Olsen-P levels were assessed in NaHCO_3_ extracts through molybdenum blue colorimetry. The P fractions were assessed using adapted methodologies adapted from Hedley et al. (1982) and Sui et al. (1999). H_2_O-Pi was extracted with deionized water, 0.5 M NaHCO_3_ was employed for NaHCO_3_-Pi extraction, 0.1 M NaOH was used for NaOH-Pi extraction, and 1 M HCl was employed for HCl-Pi extraction. Subsequently, any remaining P in the soil following the other extraction procedures was quantified through digestion with H_2_SO_4_-H_2_O_2_.

### 2.3 Statistical analysis

Statistical analyses were conducted using IBM SPSS Statistics 20 (IBM Corp., Armonk, NY, USA). One-way analysis of variance (ANOVA) with Tukey’s *post-hoc* test (*p* < 0.05) was employed to evaluate variations in shoot biomass, as well as the shoot P uptake, root biomass, root length, and Pi pools, among P fertilization treatments. Simple path analyses of the indicators affecting shoot biomass were also performed using IBM SPSS Statistics 20. Sigmaplot 10.0 (version 10.0; Systat Software Inc., San Jose, CA, USA) was used to plot charts.

## 3 Result

### 3.1 Shoot biomass and P uptake

**Figure 1:**
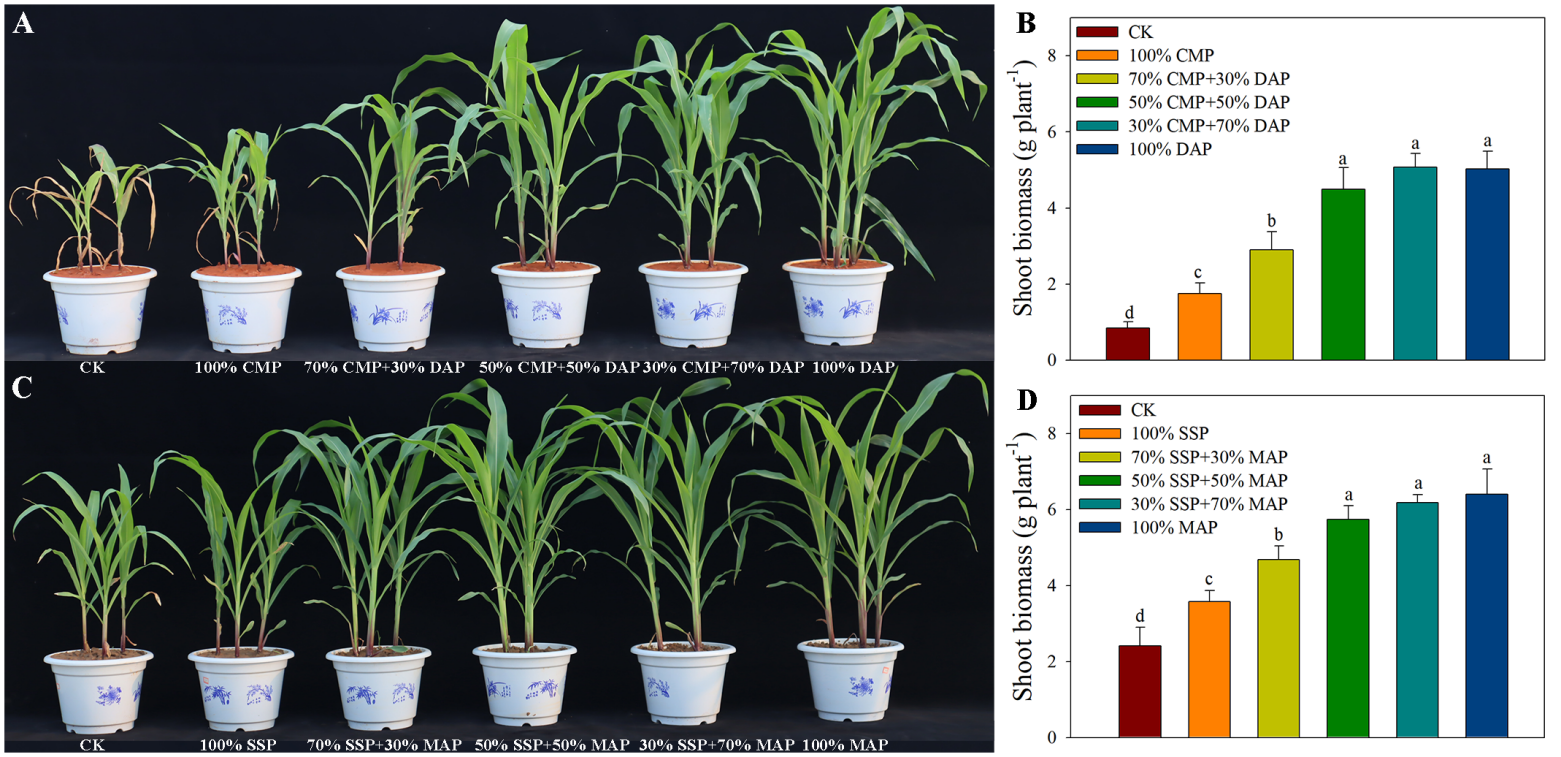
Plant growth performance **(A)** and shoot biomass **(B)** under different P fertilizer treatments in acidic soil. Plant growth performance **(C)** and shoot biomass **(D)** under different P fertilizer treatments in alkaline soil. Different lowercase letters indicate significant differences among treatments (*p* < 0.05).

There were marked crop-specific biomass responses to the CMP: DAP blends in acidic soil and SSP: MAP blends in alkaline soil (**Figure 1**). Overall, all P-fertilized plants had higher biomass than those in the CK treatment, confirming that maize was P-limited in the control soil and that the observed biomass responses were influenced by P availability. Shoot biomass was also positively correlated with the MAP and DAP proportions in the fertilizer blends. Specifically, in acidic soil, shoot biomass in the 100% DAP treatment was the highest, and although there were insignificant differences among the 50% CMP+50% DAP and 30% CMP+70% DAP treatments, both treatments exhibited significantly higher biomass than those in the 100% CMP or CK treatments. Similarly, the shoot biomass per plant in the alkaline soil treatments was ranked as follows: 100% MAP > 30% SSP+70% MAP > 50% SSP+50% MAP > 70% SSP+30% MAP > 100% SSP > CK.

**Figure 2:**
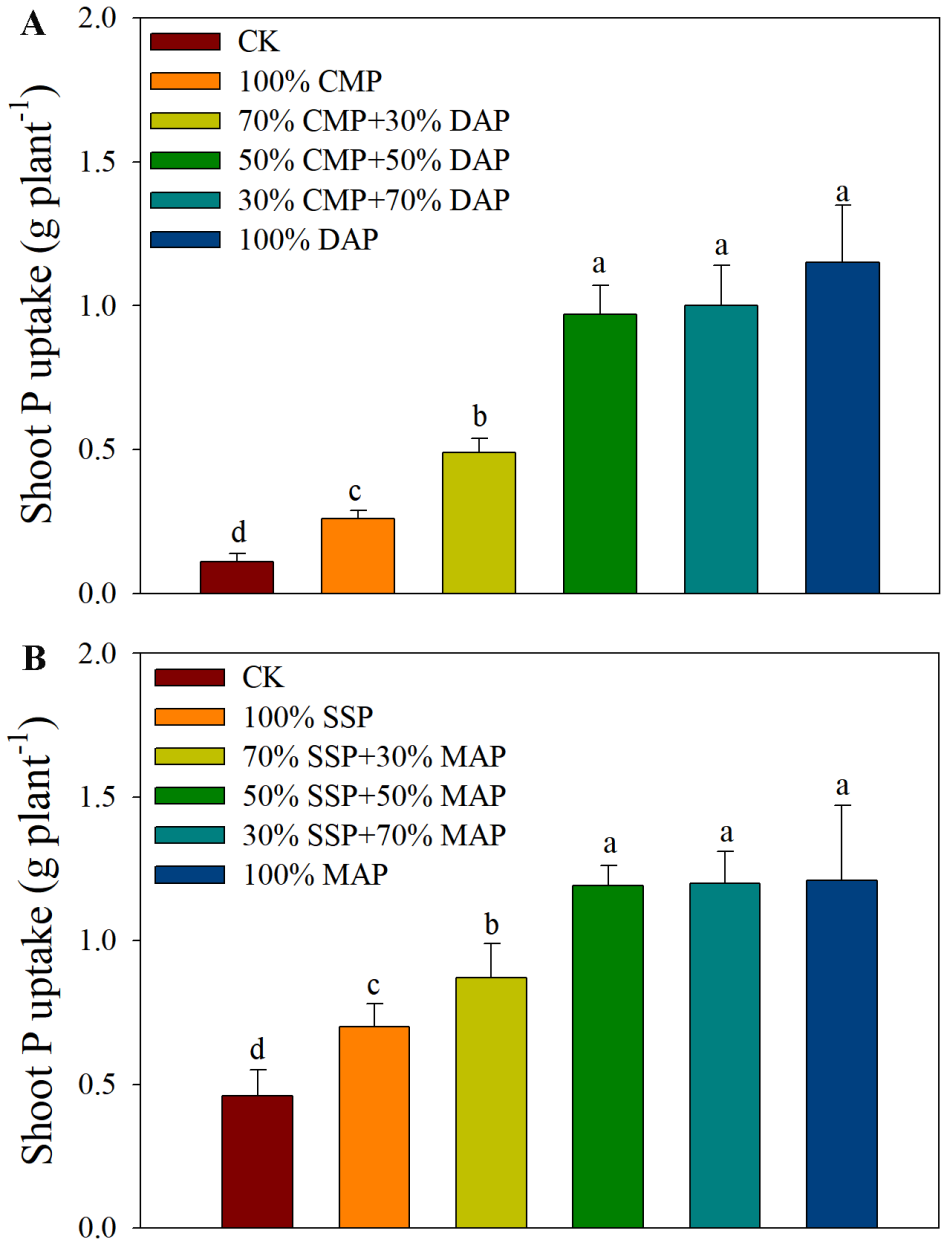
Shoot P uptake under different P fertilizer treatments in acidic **(A)** and alkaline **(B)** soil. Different lowercase letters indicate significant differences among treatments according to Duncan’s multiple range test following significant one-way ANOVA (*p* < 0.05).

Shoot P uptake was similar to shoot biomass across the P treatments (**Figure 2**). Maize in acidic soil fertilized with 100% DAP had a 77.4% higher total P uptake than maize receiving 100% CMP. In alkaline soil, maize fertilized with 100% MAP had a 42.1% higher total P uptake than maize that received 100% SSP. Interestingly, shoot P uptake in acidic soil was not significantly higher in plants that received 50% CMP + 50% DAP compared to those fertilized with 100% DAP. No difference in total P uptake was observed between maize fertilized with 50% SSP+50% MAP and 100% MAP in alkaline soil. These combined results suggest a strong correlation between P uptake and shoot biomass.

### 3.2 Root biomass and length

**Figure 3:**
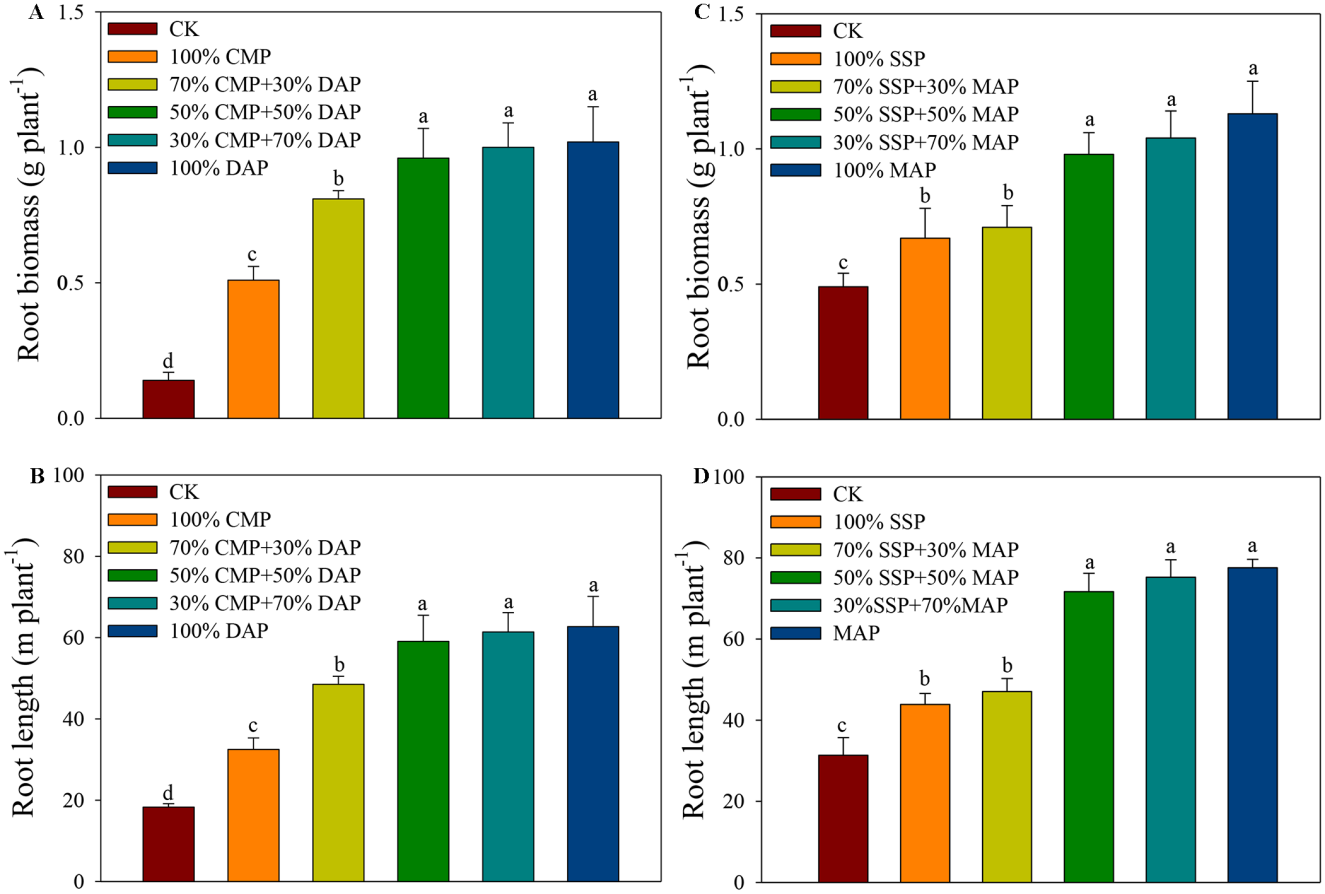
Root biomass under different P fertilizer treatments in acidic **(A)** and alkaline **(B)** soil. Root lengths under different P fertilizer treatments in acidic **(C)** and alkaline **(D)** soil. Different lowercase letters indicate significant differences among treatments according to Duncan’s multiple range test following significant one-way ANOVA (*p* < 0.05).

Soil P supply had a significant impact on root morphology regardless of soil type (acidic or alkaline). In acidic soil, the 50% CMP + 50% DAP, 30% CMP + 70% DAP, and 100% DAP treatments resulted in the highest root biomass among all treatments, with increases of 85.4%, 86.0%, and 86.3%, respectively (**Figure 3A**). These were followed by the 70% CMP + 30% DAP and 100% CMP treatments, which also showed significantly higher biomass compared to the CK treatment. In alkaline soil, the root biomass was significantly higher in the 100% MAP, 30% SSP+70% MAP, and 50% SSP+50% MAP treatments than those in the 70% SSP+30% MAP and 100% SSP treatments, with no significant differences among the 100% MAP, 30% SSP+70% MAP, and 50% SSP+50% MAP treatments (**Figure 3B**). Moreover, the root biomass in the 50% CMP+50% DAP and 100% DAP treatments in acidic soil were similar, and no significant differences were observed in root biomass between the 50% SSP+50% MAP and 100% MAP treatments in alkaline soil.

Similar to root biomass, the mean root lengths in the 50% CMP+50% DAP, 30% CMP+70% DAP, and 100% DAP treatments in acidic soil were 59.1, 61.4, and 62.7 cm, respectively, which were significantly longer than those in the 70% CMP+30% DAP and 100% CMP treatments. No significant difference in root biomass was observed between the 50% CMP+50% DAP and 100% DAP treatments. In alkaline soil, the maize root length was only 8% higher in the 100% MAP treatment than that in the 50% SSP+50% MAP treatment; however, no significant difference in length was observed between the 100% MAP and 50% SSP+50% MAP treatments (**Figures 3C, D**).

### 3.3 Soil inorganic P fractions

**Figure 4:**
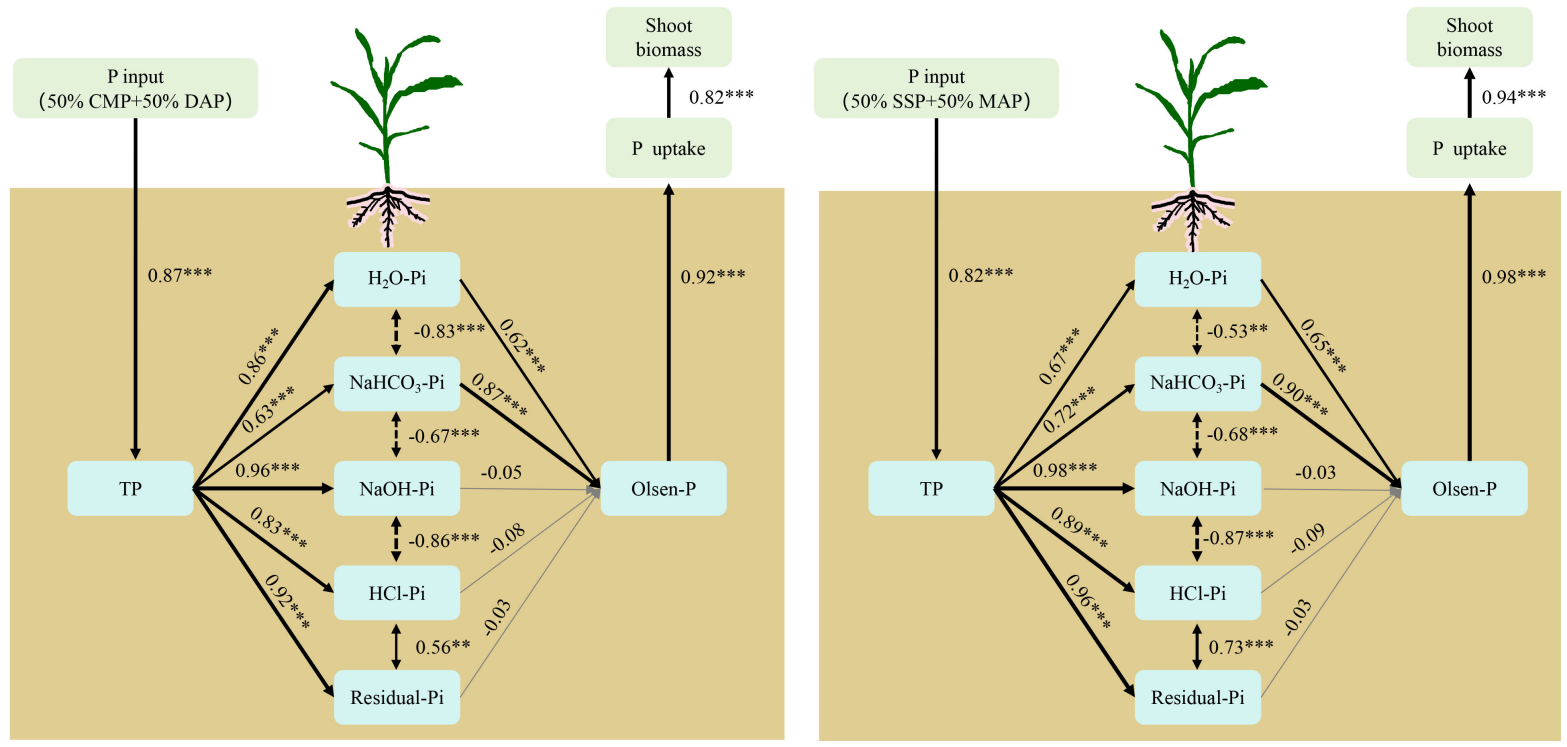
Path coefficient structural model of the relationship between shoot biomass and the other variables. Boxes indicate the variable names. Black solid and dotted arrows indicate significant positive and negative effects, respectively. Gray solid arrows indicate nonsignificant effects. Numbers adjacent to the arrows are standardized path coefficients and indicative of the effect size of the relationship. The arrow width is proportional to the strength of the path coefficient. A line with an arrowhead indicates a putative causal link between the cause (arrow base) and effect (arrow tip). ** and *** represent *p* < 0.01 and *p* < 0.001 significance levels, respectively.

The CMP: DAP blends in acidic soil and SSP: MAP blends in alkaline soil significantly increased the soil H_2_O-Pi, NaHCO_3_-Pi, NaOH-Pi, and HCl-Pi fractions when the percentage of MAP or DAP was greater than 50% (**Table 2**). In acidic soil, the soil Pi fraction in the 100% DAP treatment was the highest, with insignificant differences between the 50% CMP+50% DAP and 30% CMP+70% DAP treatments, which were significantly higher than those in the CMP and CK treatments. Compared with those in the CK treatment, the soil H_2_O-Pi, NaHCO_3_-Pi, and NaOH-Pi fractions in the 50% CMP+50% DAP treatment increased by 415%, 160.4%, and 82.8%, respectively. Similarly, the alkaline soil inorganic P fraction was the highest in the 100% MAP treatment, but no significant differences were observed between the 100% MAP and 50% SSP+50% MAP treatments. Compared with those in the CK treatment, the soil H_2_O-Pi, NaHCO_3_-Pi, and NaOH-Pi fractions in the 50% SSP+50% MAP treatment increased by 142.8%, 265.8%, and 36.5%, respectively. **Figure 4** showed a path coefficient structural model that describes the important relationships among the selected traits. The P fertilizers in both the 50% CMP+50% DAP and 50% SSP+50% MAP treatments enhanced the H_2_O-Pi fraction, which subsequently led to an increase in soil P availability. Similarly, the NaOH-Pi fraction was positively influenced by the P fertilizer inputs in the specified treatments. This fraction also contributed significantly to the overall soil P availability (Olsen-P), as indicated by the positive path coefficients.

**Table 2:** Effects of different P fertilizer treatments on soil Pi fractions based on Hedley P fraction in acidic and alkaline soil.

Soil	Treatment	H_2_O-Pi (mg kg^−1^)	NaHCO_3_-Pi (mg kg^−1^)	NaOH-Pi (mg kg^−1^)	HCl-Pi (mg kg^−1^)	Residual-P (mg kg^−1^)
Acidic soil	CK	2.64 ± 0.55c	12.49 ± 2.41b	61.50 ± 9.79c	103.88 ± 3.82c	327.04 ± 13.46b
	100% CMP	6.06 ± 1.37b	16.67 ± 2.89b	76.10 ± 12.70c	114.00 ± 4.80bc	375.58 ± 17.47ab
	70% CMP+30% DAP	9.00 ± 1.46b	21.22 ± 1.01b	86.08 ± 7.77bc	126.81 ± 8.11bc	386.57 ± 32.2ab
	50% CMP+50% DAP	13.60 ± 0.89a	32.52 ± 0.22a	112.42 ± 7.16a	129.76 ± 8.11b	437.64 ± 12.05a
	30% CMP+70% DAP	14.39 ± 1.57a	32.71 ± 2.99a	109.55 ± 5.13ab	137.83 ± 10.99ab	435.67 ± 16.08a
	100% DAP	14.49 ± 1.90a	34.64 ± 0.46a	121.28 ± 9.71a	154.71 ± 12.33a	419.48 ± 34.65a
Alkaline soil	CK	7.94 ± 1.97b	10.30 ± 0.33c	84.85 ± 5.40c	222.09 ± 4.05a	566.93 ± 32.50b
	100% SSP	7.40 ± 1.26b	12.86 ± 2.24b	102.9 ± 2.43b	223.93 ± 1.43a	559.79 ± 40.90b
	70% SSP+30% MAP	8.44 ± 1.28b	17.36 ± 1.57ab	116.08 ± 1.51a	229.09 ± 3.38a	544.58 ± 45.98b
	50% SSP+50% MAP	19.28 ± 1.41a	37.68 ± 1.94a	115.53 ± 3.36a	222.03 ± 5.73a	682.03 ± 9.73a
	30% SSP+70% MAP	19.71 ± 1.86a	36.86 ± 3.09a	113.2 ± 4.27a	222.93 ± 4.56a	684.98 ± 10.89a
	100% MAP	22.24 ± 1.37a	37.14 ± 1.67a	118.52 ± 1.26a	224.92 ± 4.70a	683.7 ± 12.98a

Lowercase letters indicate significant differences between treatments at the *p* < 0.05 level.

## 4 Discussion

### 4.1 Matching fertilizer properties with soil type for efficient P fertilizer management

Our study indicated a significant increase in total biomass under high proportions of MAP and DAP (**Figure 1**), consistent with previous studies, emphasizing the importance of balanced fertilization for optimizing crop productivity (Gong et al., 2024). The higher sensitivity of maize growth to MAP or DAP may be attributed to their use as high-concentration P fertilizers, which can provide sufficient P to help sustain early root development (Chien et al., 2011). As expected, the highest root biomass and root lengths were observed in plants subjected to the 100% DAP (acidic soil) and 100% MAP (alkaline soil) treatments. This highlights the crucial role of a well-established root system for effective P acquisition and yield potential, given the low mobility of P in the soil (Flaval et al., 2014; Hertzberger et al., 2021). The addition of high-concentration P fertilizers such as MAP or DAP is an efficient way to increase available P concentration in the soil. Moisture initiates the dissolution of fertilizer granules, allowing P to move into the soil solution due to their high water solubility (Weeks and Hettiarachchi, 2019). However, it is important to note that high-P fertilizer application does not necessarily guarantee a sustained increase in maize yield. A previous study showed that the application of low-P fertilizer (i.e., SSP or CMP) at high plant densities resulted in higher yields compared to those with high-P fertilizer applications (i.e., MAP or DAP) (Gong et al., 2022b). The high-concentration P fertilizers experience a rapid decline in water solubility due to fixation upon application to soil, limiting their bioavailability (Feng et al., 2019; Maharajan et al., 2021). Therefore, determining the optimal blend of P fertilizer is essential for promoting the conversion of low-solubility P into plant-bioavailable forms and enhancing its efficient utilization by crops.

### 4.2 The P management strategies for improving P use efficiency

Innovative approaches, such as combining high- and low-P fertilizers, can enhance soil microbial activity and facilitate the conversion of soluble P into plant-bioavailable form. The application of blended fertilizers requires careful consideration of soil properties, crop requirements, and environmental factors to maximize the benefits and promote sustainable agricultural practices. In this study, the pot experiments indicate that the 50% MAP+50% SSP (alkaline soil) and 50% DAP+50% CMP (acidic soil) treatments significantly improved P uptake, which did not differ significantly from the 100% MAP and 100% DAP treatments but were greater than that in the CK treatment, suggesting similar P uptakes as those in the 100% MAP and 100% DAP treatments.

DAP is a high-concentration P fertilizer produced by treating ammonia with phosphoric acid. Factors such as Fe and Al play significant roles in limiting plant P availability in acidic soil. DAP can contribute to higher plant P concentrations during the seedling and tillering stages due to its rapid release of phosphate (Ma et al., 2023; Ning et al., 2023). However, previous studies have shown that this rapid release can lead to P fixation through iron and aluminum oxidation in acidic soil, thereby failing to meet plant P demands during the later stages of crop growth (Chien et al., 2011; Ma et al., 2022). In contrast, MAP is a slow-acting P source that dissolves effectively in acidic soil and provides sufficient P during the later stages of crop growth (Gong et al., 2022b; Qin et al., 2022). Our results show that shoot biomass and P uptake were highest under the 100% DAP treatment but showed no significant differences when compared with the 50% CMP+50% DAP treatment-indicating a notable increase in P uptake owing to the acidulating effect of DAP on CMP.

In alkaline soils, MAP serves as an effective medium-acidity fertilizer that enhances plant P availability. Ammonium ions within MAP decrease pH levels in the rhizosphere zone, reducing phosphate fixation while promoting root proliferation–a vital reaction for improving phosphate uptake (Nunes et al., 2022). Our experimental data revealed consistently lower levels of available P in SSP compared with that of MAP and could not meet plant P demands during the early growth stages. Under similar conditions with respect to P supply, shoot biomass and P uptake in the 100% MAP treatment were significantly higher than those in the 100% SSP treatment. Surprisingly, shoot biomass and P uptake in the 100% DAP treatment did not differ significantly from those in the 50% CMP+50% DAP treatment. Therefore, the coupling of MAP and SSP provides an agronomically and economically effective way to provide the required P, as MAP can initially supply P to plants during their early development, resulting in better plant root development, moreover, the use of lower cost of SSP refers a more cost-effective alternative that could help reduce overall fertilizer expenses while still supplying adequate phosphorous for crop growth, and thus can be employed more effectively in alkaline soils (Gong et al., 2022b).

### 4.3 Blends of low- and high-P fertilizers increased soil labile and moderately labile Pi fractions

Previous studies have demonstrated the direct impact of P fertilizer inputs on soil Pi fractions (Yan et al., 2017). Our result showed the addition of chemical P fertilizers increased soil Pi with larger proportions of H_2_O-Pi, NaHCO_3_-Pi, and NaOH-Pi available to plants in the 50% CMP+50% DAP (acidic soil) and 50% SSP+50% MAP (alkaline soil) treatments. Labile P pools such as H_2_O-Pi and NaHCO_3_-Pi are direct sources for crop uptake indicating that the P applied in the 50% CMP+50% DAP (acidic soil) and 50% SSP+50% MAP (alkaline soil) treatments were retained in the more easily extracted fractions (Yan et al., 2022). Furthermore, NaOH-Pi was also a major P pool, which was enhanced by high-P fertilizer applications. Under the conditions used in this study, HCl-P_i_ was found to be an important P source for maize (in addition to H_2_O-Pi, NaHCO_3_-Pi, and NaOH-Pi) in the 50% CMP+50% DAP (acidic soil) and 50% SSP+50% MAP (alkaline soil) treatments. HCl-Pi and residual P stabilized the P with low use efficiency. Although HCl-Pi and residual P had low use efficiency, plants can enhance P uptake by modulating root morphological and physiological characteristics and by activating insoluble P through microbial activity (Shen et al., 2011; Yan et al., 2022).

In this study, the recommended P fertilizer input met the requirements for optimal crop growth, as soil P bioavailability was enhanced to promote root growth using an appropriate ratio of high- to low-P fertilizers. Enhanced root growth increases organic acid secretion and mediates rhizosphere acidification, which can activate soil P and transform insoluble P into labile P pools (H_2_O-Pi and NaHCO_3_-Pi) (Alamgir et al., 2012; Liu et al., 2023). Therefore, P fertilizer management strategies should better account for soil conditions. Additionally, optimizing soil-plant P nutrition should include a series of strategies for improving crop P uptake, reducing the excessive use of high-concentration P fertilizers, and improving low-P fertilizer concentrations.

## 5 Conclusion

The SSP: MAP blends and CMP: DAP blends were evaluated for their ability to maximize maize growth. SSP and CMP alone were insufficient to maintain maize growth. However, maize growth was comparable when up to 50% SSP substituted for MAP in acidic soil and up to 50% CMP substituted for DAP in alkaline soil. This indicates that the limitations of higher P-concentration fertilizers (i.e., MAP and DAP) can be mitigated by blending them with low-P fertilizers. Substituting SSP for MAP by up to 50% in acidic soil and CMP for DAP by up to 50% in alkaline, which can meet the crop’s P demand. These findings provide a foundation for optimizing P fertilizer types in maize production to enhance P-use efficiency. Further research could focus on optimizing these blend ratios under various soil conditions to meet maize’s P demands effectively. This optimization not only enhances P use efficiency in maize production but also promotes sustainable agriculture practices by reducing reliance on high-concentration P fertilizers, which provide a robust foundation for future studies aiming to optimize P fertilizer types in maize production, thereby contributing significantly to sustainable and efficient agricultural practices.

## Data Availability

The original contributions presented in the study are included in the article/**Supplementary Material**. Further inquiries can be directed to the corresponding author/s.
